# Spectroscopic Studies on the Biomolecular Recognition of Toluidine Blue: Key Information Towards Development of a Non-Contact, Non-Invasive Device for Oral Cancer Detection

**DOI:** 10.3389/fonc.2020.529132

**Published:** 2020-10-27

**Authors:** Soumendra Singh, Animesh Halder, Oindrila Sinha, Nilasha Chakrabarty, Tanima Chatterjee, Aniruddha Adhikari, Priya Singh, Deep Shikha, Ria Ghosh, Amrita Banerjee, Partha Pratim Das Mahapatra, Amit Mandhar, Maitree Bhattacharyya, Surajit Bose, Saleh A. Ahmed, Ahmed Alharbi, Ahmed M. Hameed, Samir Kumar Pal

**Affiliations:** ^1^ Center for Astroparticle Physics and Space Science, Bose Institute, Kolkata, India; ^2^ Technical Research Centre, S. N. Bose National Centre for Basic Sciences, Kolkata, India; ^3^ Department of Applied Optics and Photonics, University of Calcutta, Kolkata, India; ^4^ Department of Life Sciences, Presidency University, Kolkata, India; ^5^ Department of Chemical, Biological and Macromolecular Sciences, S. N. Bose National Centre for Basic Sciences, Kolkata, India; ^6^ Department of Biochemistry, University of Calcutta, Kolkata, India; ^7^ Department of MedTech R&D, Webel BCC&I Tech Incubation Center, Ezerx Health Pvt. Ltd., Kolkata, India; ^8^ Department of Dentistry, Bharat Sevashram Sangha Hospital, Kolkata, India; ^9^ Department of Oral and Maxillofacial Pathology, KSDJ Dental College and Hospital, Kolkata, India; ^10^ Department of Chemistry, Faculty of Applied Sciences, Umm Al-Qura University, Makkah, Saudi Arabia; ^11^ Chemistry Department, Faculty of Science, Assiut University, Assiut, Egypt

**Keywords:** Toluidine Blue (TB), interaction with protein and DNA, non-invasive oral cancer diagnosis, spectroscopy, oral cancer

## Abstract

Molecular interaction of aromatic dyes with biological macromolecules are important for the development of minimally invasive disease diagnostic biotechnologies. In the present work, we have used Toluidine Blue (TB) as a model dye, which is a well-known staining agent for the diagnosis of oral cancer and have studied the interaction of various biological macromolecules (protein and DNA) with the dye at different pH. Our spectroscopic studies confirm that TB interacts with Human Serum Albumin (HSA), a model protein at very high pH conditions which is very hard to achieve physiologically. On the other hand, TB significantly interacts with the DNA at physiological pH value (7.4). Our molecular studies strengthen the understanding of the Toluidine Blue staining of cancer cells, where the relative ratio of the nucleic acids is higher than the normal intracellular content. We have also developed a non-invasive, non-contact spectroscopic technique to explore the possibility of quantitatively detecting oral cancer by exploiting the interaction of TB with DNA. We have also reported development of a prototype named “Oral-O-Scope” for the detection of Oral cancer and have carried out human studies using the prototype.

## Introduction

Oral cancer is the 11^th^ most common cancer in the world (1) and has secured its place in the top 3 common cancers in India (2). Oral cancer or oral cavity cancer falls in the category of head and neck cancer which includes the malignancy of oral cavity, buccal mucosa, tongue, vermilion border of the lip, the floor of the mouth, palate, the gingiva, and also including the salivary glands, tonsils or other lymphoid tissues (3). But, the most common locations are that of the epithelia of the lining of the buccal mucosa and the lateral border of the tongue (4, 5). These types of epithelia present are squamous; hence termed as squamous cell carcinoma. Oral cancer can be developed from the de-novo or malignant transformation of Oral premalignant disorders (OPMD) (6). Oral cancer, often referred as “poor man’s disease” (7), is triggered by poor lifestyle and the major independent risk factors for Oral Squamous Cell Carcinoma (OSCC) are tobacco (through smoking and chewing), alcohol, and areca nut use whereas, poor oral hygiene, genetics, malnutrition, oral microbiome are considered as potential risk factors (8–10). Some risk factors are known for their etiopathogenic roles in oral cancer such as consumption of tobacco in a different form (chewing or smoking) and alcohol, phenol, viral (HPV, EPV, etc.), bacterial and fungal infections, electro-galvanic reaction, radiation, genetics, immunosuppression and malnutrition upregulation of oncogene, depression of tumor suppressor genes and chronic irritation from the ill-fitted denture or sharp cuspal edges of the teeth (11, 12). Apart from the confirmed cases of malignancy, some distributed cases of malignancy, arises from oral premalignant disorders (OPMD) like leukoplakia, erythroplakia, erythroleukoplakia, oral submucous fibrosis (OSMF) on the buccal mucosa, tongue and the floor of the mouth, palate, etc. In India and South East Asia malignant transformation of OSF to squamous cell carcinoma (SCC) has been estimated to be between 2 and 8% and malignant transformation rates of oral leukoplakia from 0.13 to 17.5%, whereas 5-year cumulative malignant transformation range from 1.2 to 14.5% (13). If these can be detected through careful visual screening in the early stage where these oral cancers have not propagated to the lymph node yet, the patient can be treated with a high survival rate (14–16).

Toluidine Blue/Toluidine Blue O/Basic Blue 17/Tolonium chloride/Butene chloride (Molecular Formula:C_15_H_16_C_l_N_3_S) is a phenothiazine cationic basic, hence acidophilic dye. By exploiting its metachromatic properties, it is expected to bind to acidic components of the tissues like sulfates, carboxylates, and phosphates. It interacts with macromolecules and provides us with a unique yet simple visual molecular recognition technique by staining nucleic acid and polysaccharides. This compound fulfills the criteria of being a suitably charged stain as it cannot cross the plasma membrane of the cells, thus finding its application in *in vivo* staining. It found its first application in *in situ* detection of cervical cancer in 1963 (17). Similarly for the patients, with oral lesions, cysts or other neoplasms or for the patients with potential malignancy in their aerodigestive tracts, a 1–2% rinse of the Toluidine Blue (TB) dye or an aqueous based or a weak acid based application will ensure that the dye only retains in those malignant or the potentially malignant tissues (and also the non-specific retention of the dye in dead tissues such as on the upper surface of the tongue. The relative staining of the tissues which provides almost a gradient of colors in the bluish-purple range in the localized malignancies is indicative of the presence of the malignancy and gives us a qualitative or a visual idea of the stages of the carcinoma. Since TB is a basic and a cationic dye it will have an affinity towards the negatively charged components of the tissues such as DNA and RNA. The malignant epithelia of the mouth contain more amounts of nucleic acids along with wider intracellular channels which help in an increased penetration and retention of the dye than the non-malignant tissues. Thus, after rinsing off the buccal cavity, only the malignant areas would retain the stain and the non-malignant parts would eject out the bulky stain which will not be able to cross its plasma membranes and interact with the macromolecules (16, 18). Although the TB staining is found to be effective in the screening method for the recommendation for biopsy test to diagnose the oral cancer, frequent false positive and failure of quantitative estimation of the degree of cancer are found to be limitations of the technique. The conventional technique of TB staining of oral lesion and thereafter visual and colorimetric evaluation for detection of carcinoma is a highly qualitative process largely dependent on the experience of the practitioner, and also leads to false positives in many cases.

Despite the widespread developments in medical therapeutic techniques, the survival rate of Oral Squamous Cell Carcinoma (OSCC) in last 5 year is unchanged globally (19, 20). This can be attributed to delayed diagnosis, owing to absence of appropriate diagnostic tools for early and quick determination of oral malignancy in human cells. Tissue cell biopsy remains to be gold standard for OSCC identification. In recent developments, few optical light-based imaging devices have been reported and few of them have been made commercially available also (19). Most of them use either autofluorescence or chemiluminescence properties of live cells to represent visual gradient between the images of healthy cells and malignant cells. Although these medical advancements have shown great potential, but some factors prevent the extensive use of such technology. The problem of relying on colorimetry and qualitative nature of analysis restricts the existing devices with accuracy and measuring level of malignancy in numerical values. The molecular level identification of interactions of cells with other staining agents also remains questionable.

In our work, we are interested in exploring the basic photochemistry behind the application mentioned above and simultaneously explore the possibility of quantitatively detecting oral cancer *via* spectroscopic methods exploiting the interaction of TB with DNA. In a recent study, it is shown that absorption spectrum of TB can fitted in to different bands of six different aggregation species simultaneously present in the sample under investigation (21, 22). The overall TB spectrum may be mainly attributed to the H-type aggregation, although some of the species also show the J type bands with distinct spectrum band. The interest is to recheck the evidences of interaction between the dye and the macromolecules such as DNA and protein. Though it has already been reported in the literature with stress that the dye must interact with the nucleic material of the virtue of its anionic and acidic nature (18), the strong confirmation of whether this dye interacts with protein at different pH is still unrevealed and this area will be focused in our work by exploiting the spectroscopic or absorption properties of the chosen model protein and the dye. The understanding of pH dependent interaction of TB will enable us to exploit its character in a direction where we can use it for oral cancer detection. We are also reporting the development of a prototype for malignant cell detection utilizing the fundamentals of reflection spectroscopy. The prototype has also been shown to be producing significantly accurate data when used in patients for preliminary clinical studies. The prototype has shown potential to successfully measure malignancy in small number of human subjects. Although, extensive clinical trials need to be undertaken in future for the data to be statistically significant. We hope that toluidine blue staining method which was considered unreliable for its classical dependence on the experience of practitioners (19), would gain significant importance with its complementary use with Oral-O-Scope.

## Materials and Methods

Chemicals: Human Serum Albumin (HSA), Calf Thymus DNA, Sodium Chloride (NaCl), Potassium Chloride (KCl), Potassium Dihydrogen Phosphate (KH_2_PO_4_), Disodium Hydrogen Phosphate (Na_2_HPO_4_) and Glycine were obtained from Sigma-Aldrich (St. Louis, USA). Toluidine Blue powder was obtained from Sisco Research Laboratories Pvt. Ltd. All the reagents were of analytical grade and were used as received.

### Methods of Sample Preparation

Phosphate Buffer Saline (PBS, pH 7.4) was prepared by dissolving 0.13 M of NaCl, 2.7 mM of KCl, 1.4 mM of KH_2_ PO_4_, 0.01 M Na_2_HPO_4_ in 1 liter millipore water. Glycine- Hydrochloric acid buffer (0.1M, pH 2) was prepared by dissolving 0.1 M of Glycine and 0.02 M HCl into water. Glycine-NaOH buffer (pH 11) was prepared by dissolving 0.01 M Glycine and 0.01 M NaOH in water. A stock concentration of 42 mM TB was prepared in distilled water. For absorption measurement, the stock solution of TB was diluted to 100X and 200X times for further use. Stock solutions of HSA and DNA were prepared in 50 mM phosphate buffer of pH 7.4. A stock solution of TB in water was prepared daily for the spectroscopic measurements. To study the interaction of HSA with TB, HSA was used in the increasing ratio of 1:1, 1:10, 1:25, 1:50, and 1:100. To study the effect of pH in the interaction of TB and HSA, HSA was dissolved in buffers of different pH, keeping the concentration of TB constant.

### Characterization Techniques

The absorption measurements were performed with Shimadzu UV-2450 UV-Visible Spectrophotometer. We have used the buffer at various pH to create the baseline for absorbance measurement of the corresponding spectra. All the picosecond resolved fluorescence transients were measured by using commercially available time-correlated single-photon counting (TCSPC) setup with MCP-PMT from Edinburgh instrument, U.K. (instrument response function (IRF) of ∼80 ps) using a 409 nm excitation laser source. The details of the time-resolved fluorescence setup are identical to the previously reported article (23–28). A quartz cuvette of path length 1 cm was used for all the optical measurements. To estimate the Forster resonance energy transfer efficiency of the donor (EtBr) to the acceptor (TB) and hence to determine the donor-acceptor pairs we have followed the previously reported methodology. The donor-acceptor distance (r) can be calculated using the formula r_6_ = [R_0_ 6 (1−E)/E], where R_0_ is the Forster’s distance and E is the efficiency of energy transfer. Here, the efficiency of energy transfer (E) is calculated from the lifetimes of the donor in the absence and presence of acceptors (τ _D_ and τ _DA_) using the formula: E = 1-τ _DA_/τ _D_.

#### Cell Culture

Human lung carcinoma (A549) cell and human embryonic kidney (HEK293) cells were purchased from National Center for Cell Science, Pune, India. Cells were cultured in DMEM media (pH 7.4) supplemented with 10% FBS and antibiotic-antimycotic solution 100× (containing 10,000 units penicillin, 10 mg streptomycin, and 25 µg amphotericin B per ml in 0.9% normal saline). The cell lines were maintained at 37°C in an air-jacketed 5% CO_2_ incubator and were routinely passaged.

#### Toluidine Blue Staining

All the cells were separately plated at a density of ~100 cells in a 96-well plate. After 24 h incubation, 2 µM Toluidine blue dye was treated for 30 mins at 37°C. Then the cells were washed with 1X Phosphate Saline Buffer and OD was taken at 640 nm. The same procedure was repeated after the 2^nd^ wash with 1X PBS.

#### Confocal Microscopy

A549 cells and HEK cells were seeded on the cover slip and grown in DMEM media. Cells were fixed for 30 min with 4% paraformaldehyde and subjected to confocal microscopic studies. Cells were then incubated with 2 µM Toluidine blue for 15 m, washed twice with 1x PBS, and incubated with EtBr for 15 m. After washing in the same procedure, cells were mounted with anti-quenching agent *n*-propyl gallate for microscopic slide preparation and detected by confocal fluorescence microscope.

#### Spectrometric Device for Detection of Oral Cancer (Oral-O-Scope) (Patent Application Number: TEMP/E-1/34402/2019-KOL)

**Figure 1:**
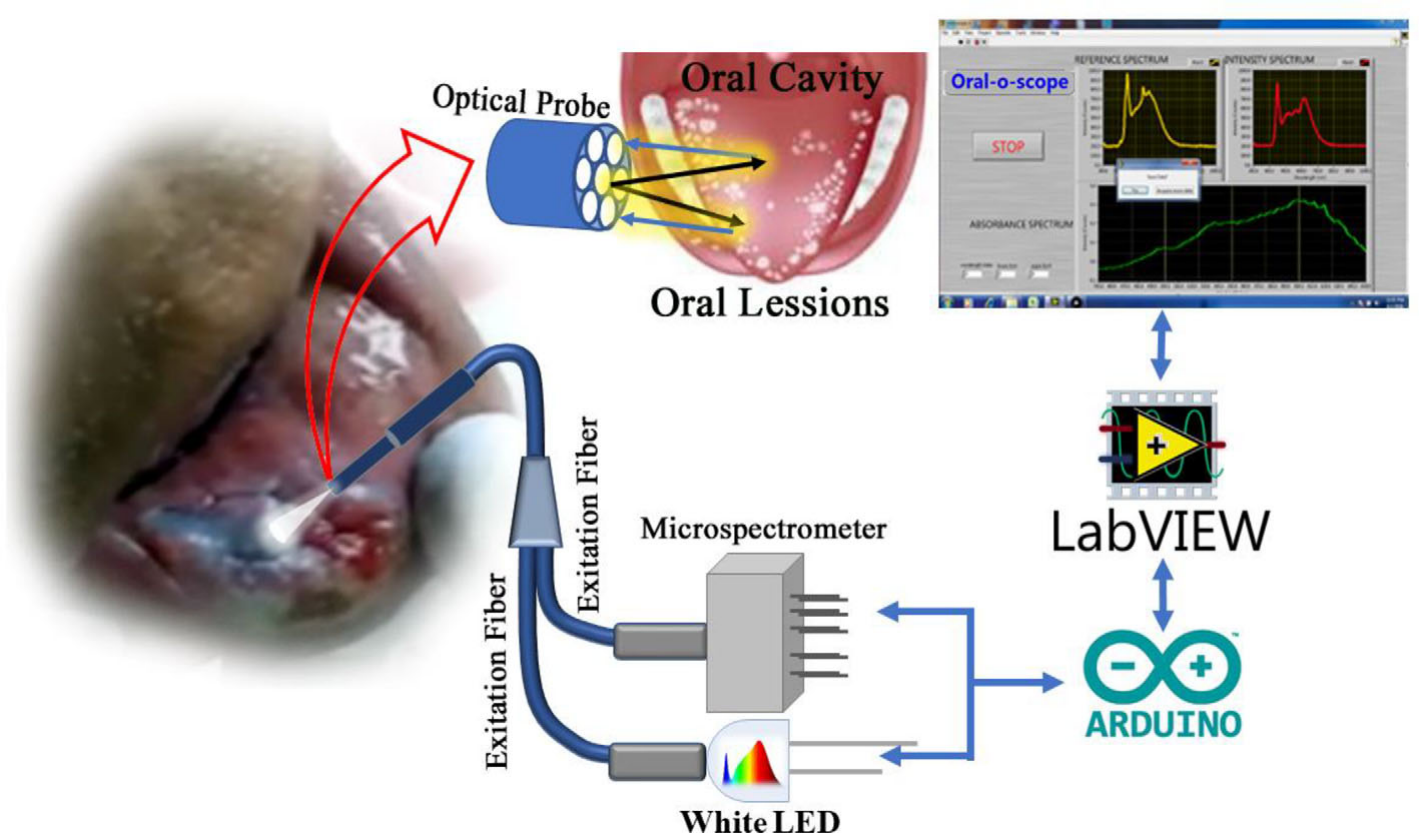
Schematic diagram of the components in the “Oral-O-Scope”. Light from a 3 W LED source is allowed to incident in the Toluidine Blue (TB) stained oral lesion and collect the difused reflected light through a 6:1 fiber optic bundle to a microspectrometer mounted on a arduino platform. The spectroscopic signal is analysed threough a self-developed Labview based software.

Reflection spectrometry was used for the development of a device capable for non-invasive and non-contact development of oral cancer cells. This device consists of primarily a light source, a spectrograph, and a lab grade reflectance probe. A 3-watt LED light available in the local market was used to collect light and incident on the cells under test. Appropriate power supply was provided using a 5 Volt AC-DC adapter. The retro-reflected light from the sample was collected and fed to a spectrograph purchased from Pure Engineering. The optical signals after the successful analysis was passed on to a computer *via* a micro controller (Arduino Uno). An in-house designed software acquires the necessary information and produces the plot in real time. A lab grade diffuse reflectance probe (Ocean Optics, Florida) having six illumination fibers around one acquisition fiber was used to transmit the light from the LED source and to accept the diffuse optical signal from the sample and send the signal to the spectrometer respectively. The schematic of the developed device is shown in **Figure 1**. For the use of the developed device on human subject, informed consent was obtained from the patients prior to the application of TB and data acquisition. Removal of oral debris was achieved by rinsing with water for 30 s. This was followed by a 1% acetic acid rinse for 20 s. Finally, the oral cavity was rinsed with (1%W/W) TB for full 30 s. The mechanically retained TB stain was eliminated using 1% acetic acid rinse for 20 s (26–28). The clinical study was carried out in 20 patients after obtaining ethical clearance (ADCH/OP-513/16-11/906) issued by “The Ethical Committee”, Awadh Dental College & Hospital, Jamshedpur, India. All methods in this study were carried out strictly following guidelines and regulations set by the ethical committee.

#### Software of the Device

A GUI based software was developed to acquire signals and plot them in real time and quickly arrive at the decision of screening the patient. The computer connects to the developed device *via* a USB and listens to the interface for incoming data. The acquired data will be an array of the intensity values at various wavelength after necessary calibration. The calibration constant was provided by the manufacturer. The software developed in LabVIEW platform, is simple, intuitive and needs no trained manpower to operate.

#### Work Flow

**Figure 2:**
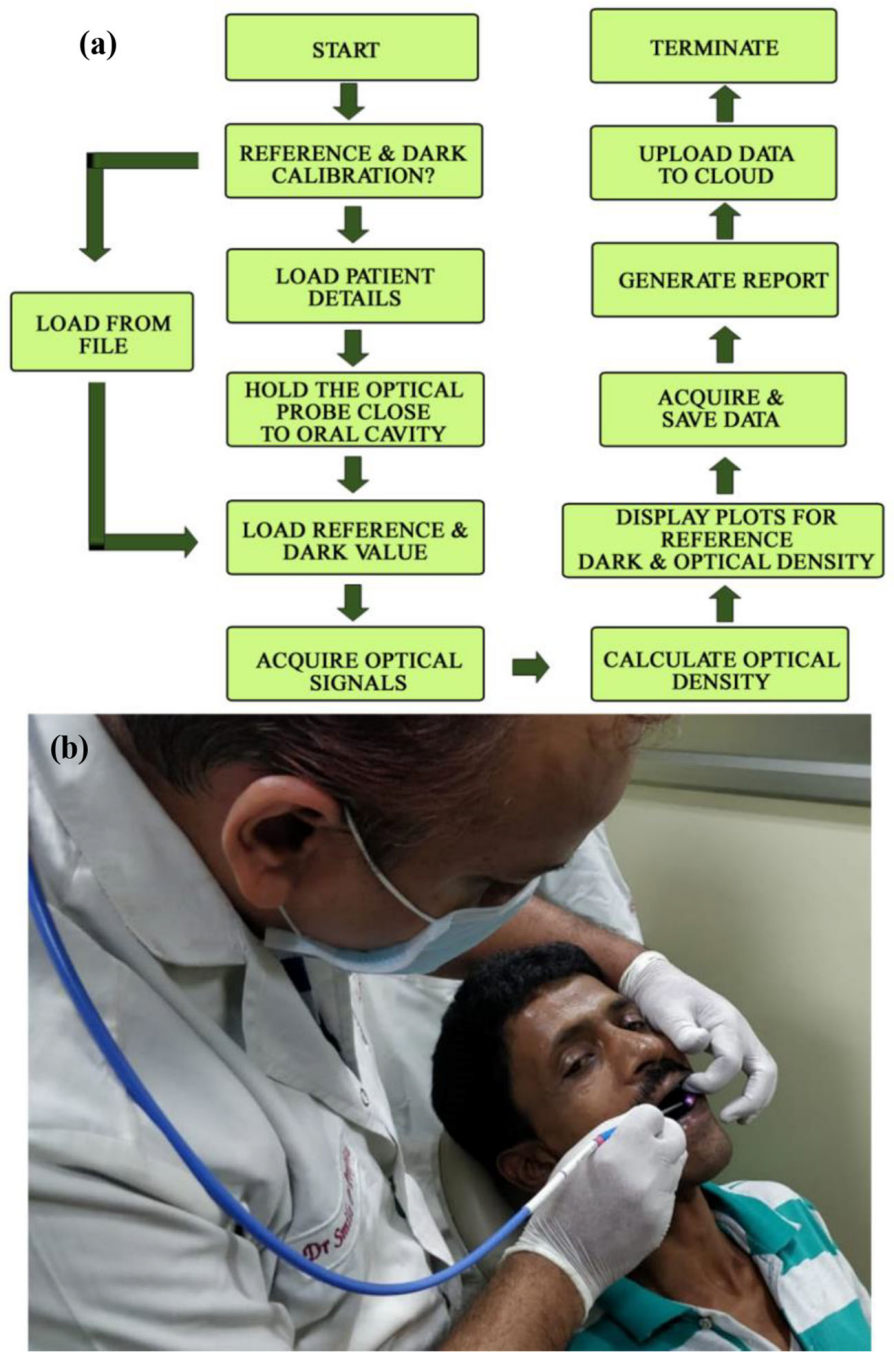
The work flow of the developed Oral-O-Scope is shown in **(A)**. **(B)** shows actual procedure of using the device by a dentist on a human subject. Live details are shown in a supplementary video (Informed consent from all human subjects and participants to publish their respective photo/video in an open access journal was obtained).

The working principle of the developed instrument “Oral-O-Scope” has been demonstrated in **Figure 2**. After initiation of the GUI, it performs its “homework” about the availability and health of the connected equipment. After successful establishment of the link to the instrument, the software asks the user to perform at reference and dark value determination procedure. This is a very important step to arrive at the Optical Density guided by Beer Lambert’s law. The reference and dark values can also be a preloaded set of value stored on the hard drive as text files or can be instantaneously provided. The users are then provided with the live intensity (pixel counts) window, stored values for reference intensity and the Optical density window. The reflectance probe was held close to the oral cavity of the patient. The light incident (400–900 nm) on the tissue of the oral cavity illuminates the lesion under test and the retro-reflected is collected by the center fiber and is taken to spectrograph. All obtained graphs were fitted with multiple Gaussian peaks to evaluate the individual contribution of each component in the plot. The process of fitting was followed according to one of our earlier work (29). Each component of the graph corresponds to certain form of molecular TB or some tissue spectroscopic signature. It was observed that the spectra obtained from malignant lesions showed a distinctly different pattern than the normal cells. Multiple sets of data were acquired and averaged to produce a steady trace free from errors that may arise from hand or subject shaking during the time of data acquisition. The software automatically decides to acquire the valid coming from the muscle an ignores any spurious signal.

## Results and Discussions

**Figure 3:**
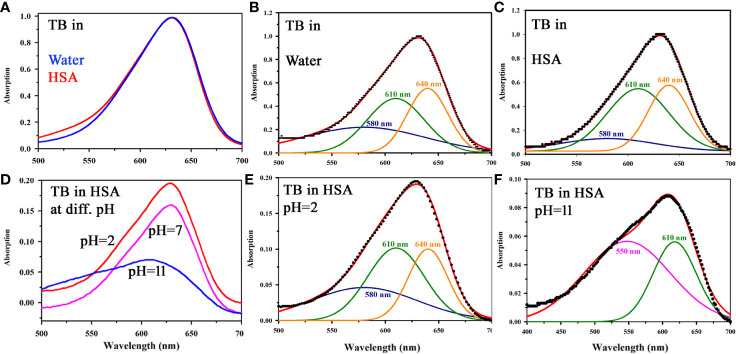
**(A)** Absorption spectra showing the interaction of TB with HSA in a ratio (1:1) in water. **(B)** Deconvoluted spectrum of TB in water **(C)** Deconvoluted spectrum of TB in complexation with HSA (in a ratio of 1:1) showing the presence of monomer (610 nm) along with the dimeric form (580 and 640 nm). **(D)** Steady state absorption spectra of TB in presence of HSA in a ratio of 1:1 at pH 2, 7, and 11 **(E)** The deconvoluted spectrum of TB in complexation with HSA (in a ratio of 1:1) at pH 2 **(F)** The deconvoluted spectrum of TB in complexation with HSA (in a ratio of 1:1) at pH 11.

Although the cationic dye TB is being used as screening stain for the diagnosis of oral cancer for long time (30), a systematic study on the interaction of the dye with biological macromolecules is sparse in the literature. In a molecular simulation study followed by X-ray crystallographic investigation, it is shown that TB in solution may remain in six different aggregated forms (21, 22). We have observed that the aggregation behavior of the dye TB depends on the biological macromolecules and their conformation. **Figure 3A** shows absorption spectrum of TB in water and in human serum albumin (HSA protein at pH 7). Even with higher concentration of HSA (1:100) no observable change in the TB spectra was noticed. Spectral deconvolution of TB in water is shown in the **Figure 3B** were presence of monomer (610 nm) along with some dimer (580 and 640 nm) are evident. **Figure 3C** shows minor but distinct difference of TB deconvoluted spectrum in HSA at pH 7 compared to that in water. The absorption spectral characteristics of TB in HSA solution at various pH conditions are shown in **Figure 3D**. While deconvoluted spectrum of TB-HSA in pH 2 (**Figure 3E**) is consistent with that of pH 7 **Figure 3C**, the spectrum at pH 11 (**Figure 3F**) is distinctly different as the protein undergoes structural denaturation revealing unprecedented absence of dimeric form (580 and 640 nm) rather indicate the presence of trimeric species (550 nm). As high pH conditions are rare in biological systems (31) and it is almost impossible to achieve in oral cavities this study gives us the basis for using the dye as an oral cancer detection probe. As the pH condition in cancer cells are deregulated and lowered, TB will not be able to bind with protein in cancer cells in normal circumstances. However, denatured protein is shown to have binding affinity, which may have some physiological significance. For example, it was observed that filiform papillae, when exposed to the toluidine blue, always retain the dye. Although the mechanism was not clear, it might be related to a high protein-synthesis rate (32). In a recent study it is also reported the presence of denatured sensory proteins in the filiform papillae (33).

**Figure 4:**
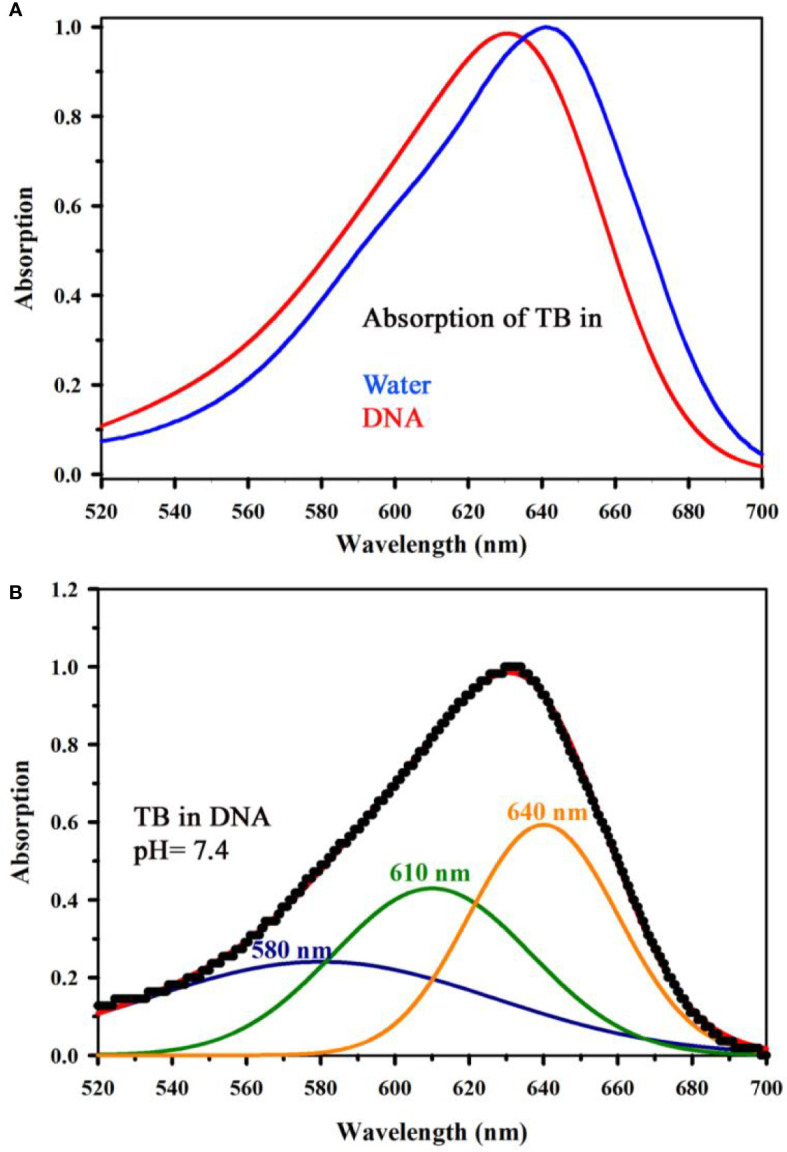
**(A)** Absorption spectrum showing the interaction of Toluidine Blue (TB) with Genomic DNA (from Calf Thymus) (in a ratio of 1:1) along with the spectrum of TB in water has also been shown for comparative analysis of TB in water and in complexation with DNA. **(B)** shows deconvoluted spectrum of TB complexed with DNA, where the signature of monomeric form of TB (610 nm) along with the dimeric forms are visible (580, 640 nm).

**Figure 5:**
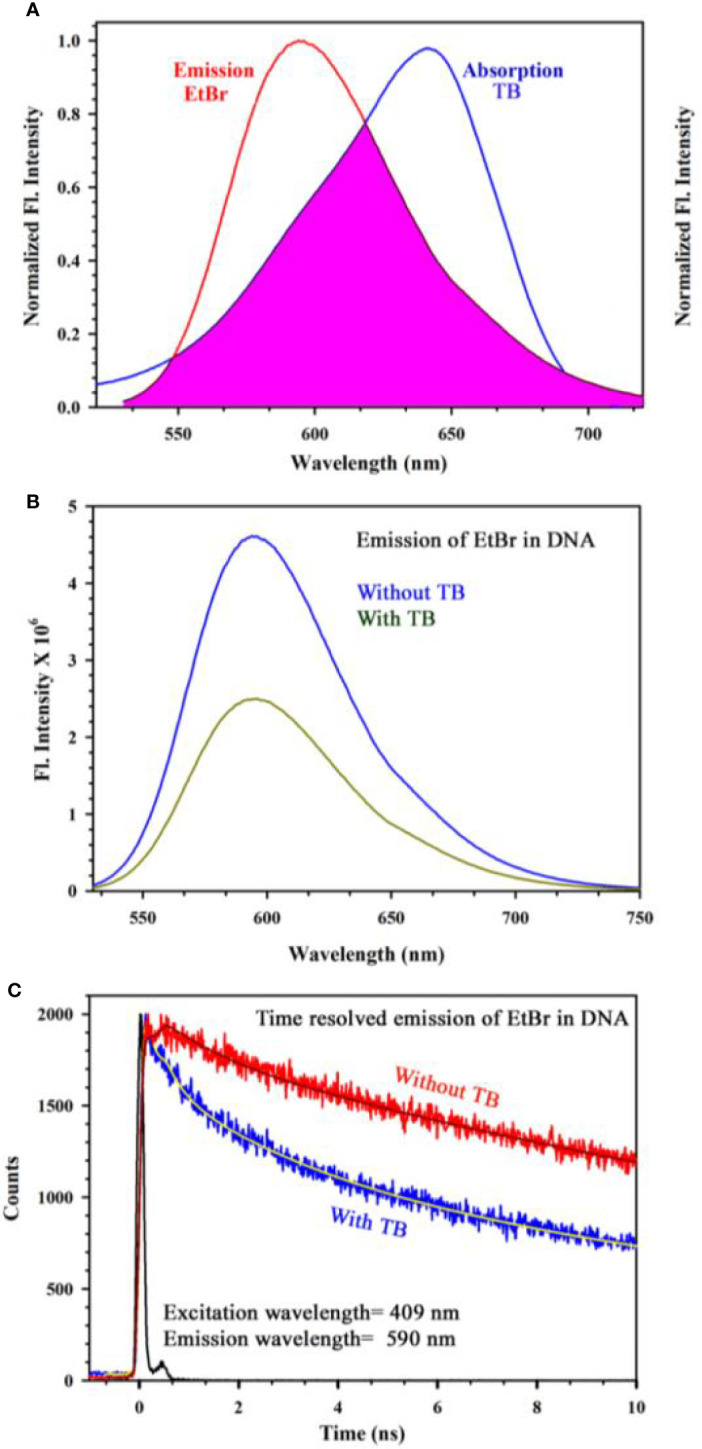
**(A)** Steady state emission spectra of EtBr and absorption spectra of TB are shown. An overlapping zone between the emission of EtBr and TB has been indicated as the pink shaded zone. **(B)** Steady state emission spectra of EtBr (donor) in absence and presence of the acceptor molecule (TB) **(C)** The picosecond resolved fluorescence transients of the donor (EtBr) in absence and presence of the acceptor (TB) are shown.

**Figure 4A** shows absorption spectrum of Toluidine Blue (TB) in Genomic DNA (from Calf Thymus). The spectrum of TB in water is also shown for comparison. An apparent shift in the TB-DNA spectrum compared to that in water is evident. The absorption peak of TB in water is at 630 nm which is shifted to 640 nm on binding to calf thymus DNA. In Fig 4B, the absorption spectra show the binding between TB and calf thymus DNA with an absorption maximum at 636 nm. Panel (**Figure 4B**) shows deconvoluted spectrum of TB-DNA, where the presence of monomeric form of TB (610 nm) along with dimeric form are evident (580, 640 nm). This establishes that DNA interacts with the dye in a specific manner consistent with reported literature (34) and offers opportunity to exploit the property to identify malignant cells using the spectroscopic technique. As malignant cells have more amount of nucleic acid as it is highly proliferating, therefore, the cells are expected to be strongly stained with TB (32). In order to study the specific molecular recognition of TB by the genomic DNA, we have performed the FRET studies to investigate the energy transfer from EtBr (a known intercalary dye for DNA) to TB in the condensate. **Figure 5A** shows the emission spectra of EtBr overlaps with the absorption spectrum of TB. The fluorescence transient of EtBr is quenched in presence of TB as shown in **Figure 5B**. The efficiency of energy transfer in the above system is found to be 45% from the temporal fluorescence decay (**Figure 5C**). The distance between the donor and acceptor was calculated to be 36 Angstrom. Thus, the experimental finding indicates that EtBr and TB can be intercalated simultaneously maintaining a distance of about 10 base pairs (21). It has to be noted that, higher concentration in TB compromises structural integrity of the DNA and eventually the DNA-TB complex precipitates. These results help us to conclude that TB and EtBr both bind the DNA. Hence, TB stain can be a potential marker for increase in nuclear materials which is the characteristic of malignant cells.

**Figure 6:**
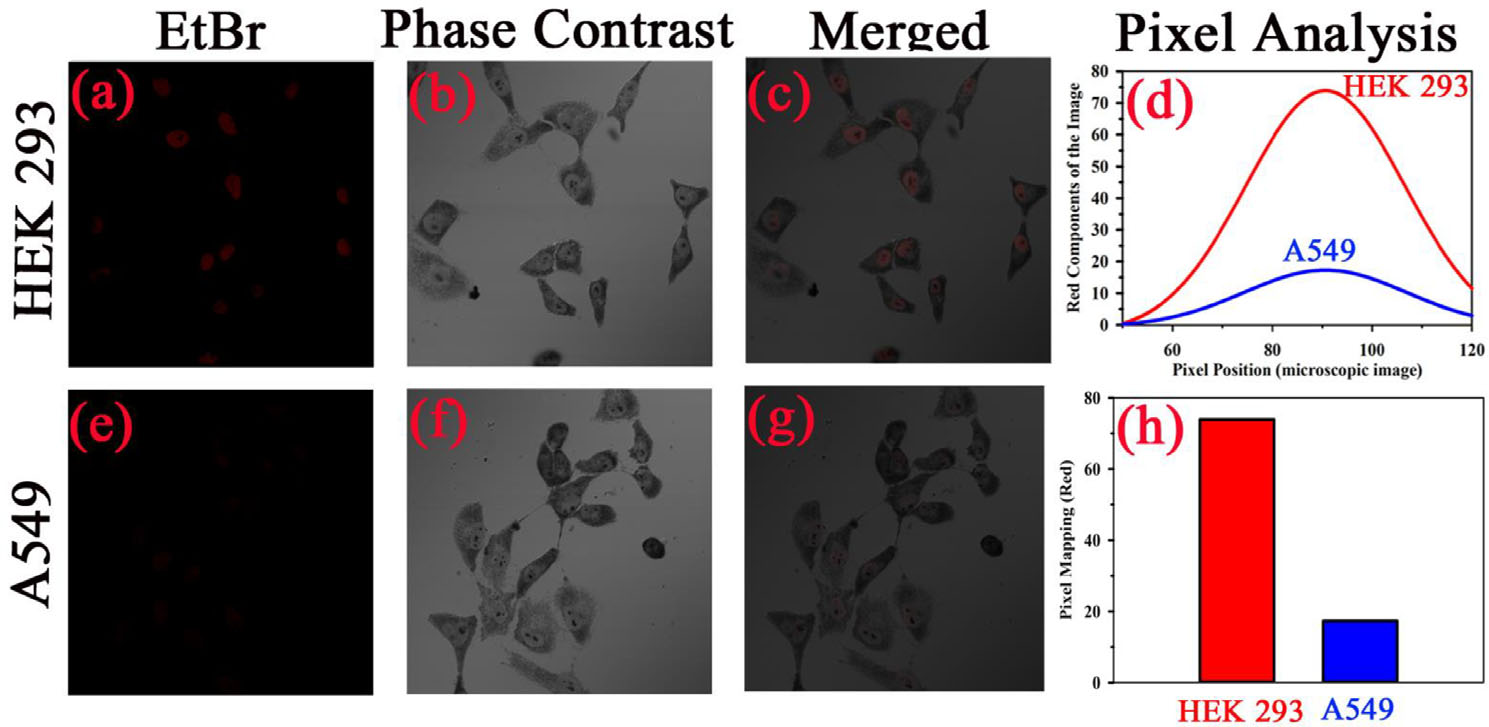
Specific localization of TB in nucleus of malignant cells resulting into the increased FRET efficiency with EtBr. Phase contrast microscopic image human embryonic kidney (HEK293) cells and lung cancer cell (A549) is shown in **(B, F)**, respectively. The fluorescence images of the cell lines with Ethidium bromide (Etbr) labelling in absence and presence of toluidine blue (TB) are shown in **(A, E)**, respectively. The fluorescence quenching of the EtBr stained nucleus in presence of TB is evident. **(C, G)** shows the merged image. A quantitative estimation from red component in the nucleus images from RGB analysis is shown in **(D)**. **(H)** shows the fluorescence intensity of EtBr after TB staining in both cells as per RGB analysis.

**Figure 7:**
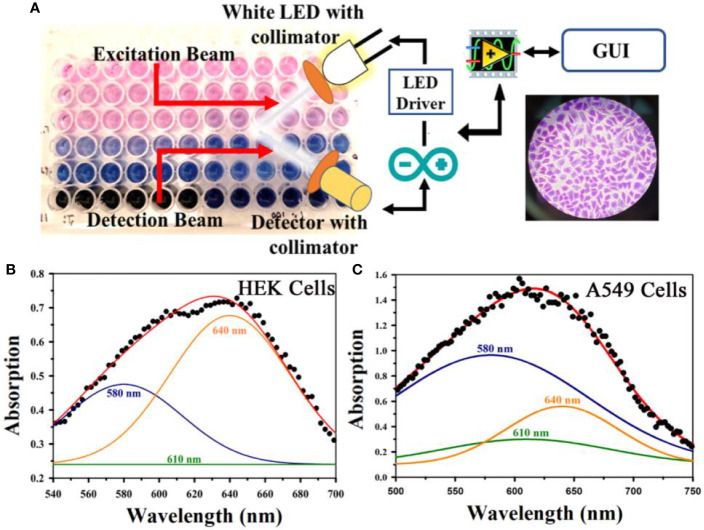
**(A)** A representative photographic image of 96 well plate containing either normal embryonic kidney (HEK) cell or lung cancer (A549) cell lines with TB staining of different concentrations is shown. The arrangement of spectroscopic investigation and microscopic image of TB stained A549 cell lines is also shown. Deconvoluted TB absorption spectra for HEK and A549 are shown in **(B, C)**. Note that Monomeric TB form (610 nm) is only present in the cancer cell lines in contrast to its normal counterpart.

After conducting the biophysical study on the specific interaction of TB with DNA, we further investigated the effect of TB on cell lines. As our ultimate goal was to evaluate the specificity of TB in staining of oral cancer cells, we choose epithelial cells for ex vivo evaluation. For our experiment, we have selected human embryonic kidney (HEK 293) cell line as control and A549 lung cancer cell lines as malignant cells (**Figure 6**). Phase contrast microscopic image of HEK and lung cancer cell lines (A549) is shown in in the **Figures 6B, F**. The fluorescence images of the HEK and A549 cell lines with Ethidium bromide (EtBr) labelling are shown in the **Figures 6A, E**. A quantitative estimation from red component in the nucleus images from RGB analysis is shown in the **Figure 6D**. A marked decrease in the red fluorescence of EtBr is observed which indicates increased localization of TB in malignant cells and thus higher amount of quenching of EtBr emission. The authors would like to point out that the focus of this study was to examine the co-localization of TB and EtBr in the nucleus of the cell. A FRET between EtBr and TB dictates that they are in close proximity to each other. This further strengthens our proposition of TB being a predominant staining agent specific for malignant cells. **Figure 7A** depicts photographic image of 96 well plate containing either normal HEK 293 cells or A549 cells with TB staining of different concentrations. Only tissues with confirmed case of malignancy were chosen to perform experiments in the laboratory as permitted by the ethical committee. The arrangement of spectroscopic investigation and microscopic image of TB stained A549 cell lines is also shown. Deconvoluted TB absorption spectra for HEK 293 and A549 are shown in **Figures 7B, C**. Note that monomeric TB form (610 nm) is only present in the cancer cell lines in contrast to its normal counterpart. The experimental observation clearly depicts that presence of 610 nm spectral line may be accompanied with dimeric or trimeric TB species is indicative of malignant cell. These forms of TB can be characterized by the individual contribution of deconvoluted peaks in amplitude and width (area under the curve) obtained from the acquired data

**Figure 8:**
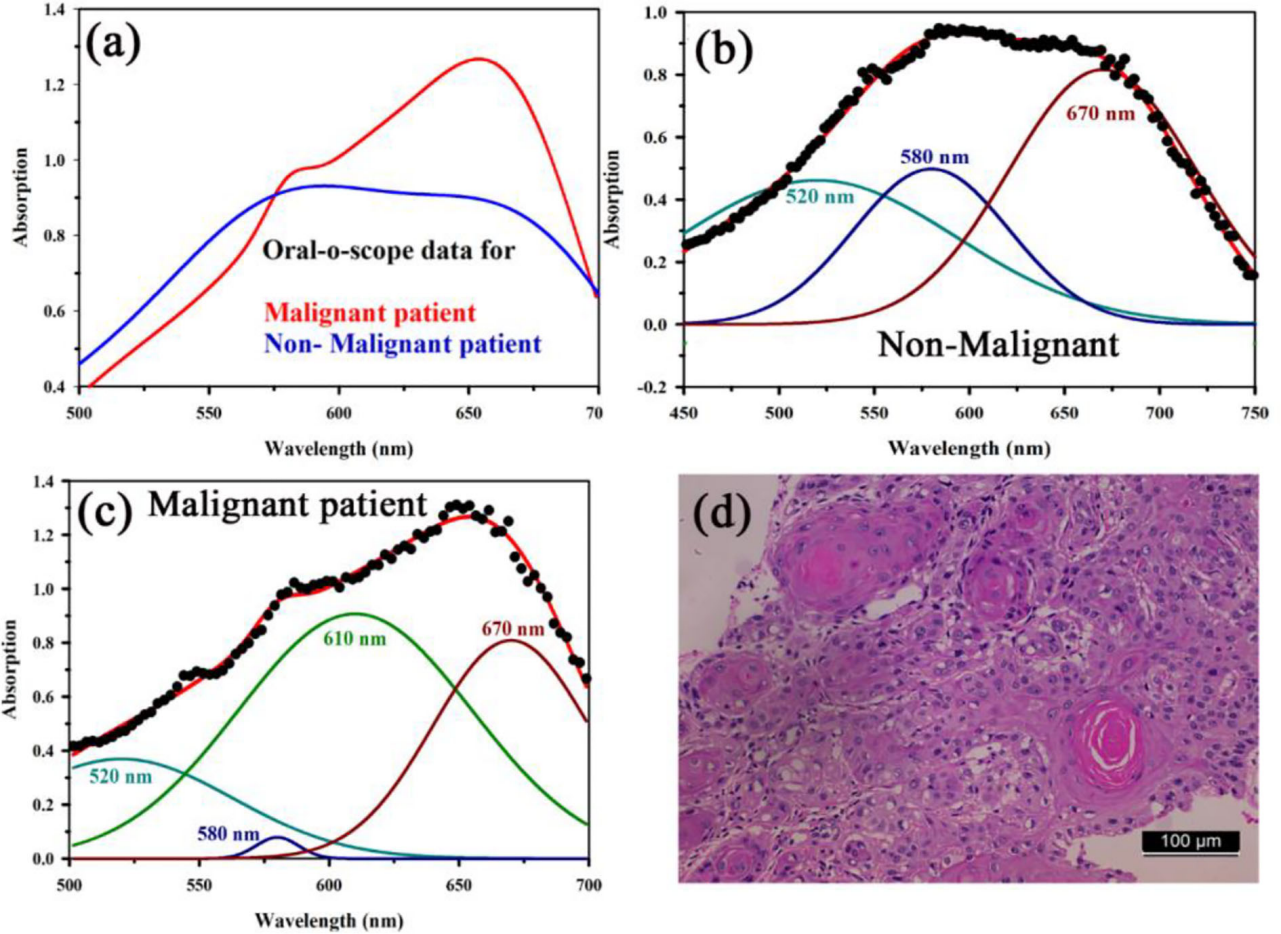
**(A)** Absorption spectra acquired through “Oral-O-Scope” during a clinical trial on malignant and non-malignant human subjects. The spectral deconvolution as shown in panels **(B, C)** clearly reveals absence of monomeric form of TB (610 nm) in non-malignant lesion **(B)** compared to that in malignant lesion **(C)**. The presence of monomeric TB associated with tetrameric forms (520, 580 nm, and 670) is distinctly evident in the malignant tissue. Corresponding confirmatory biopsy (histopathological slide) image of the TB stained tissue from the malignant subject is shown in **(D)**.

**Figure 9:**
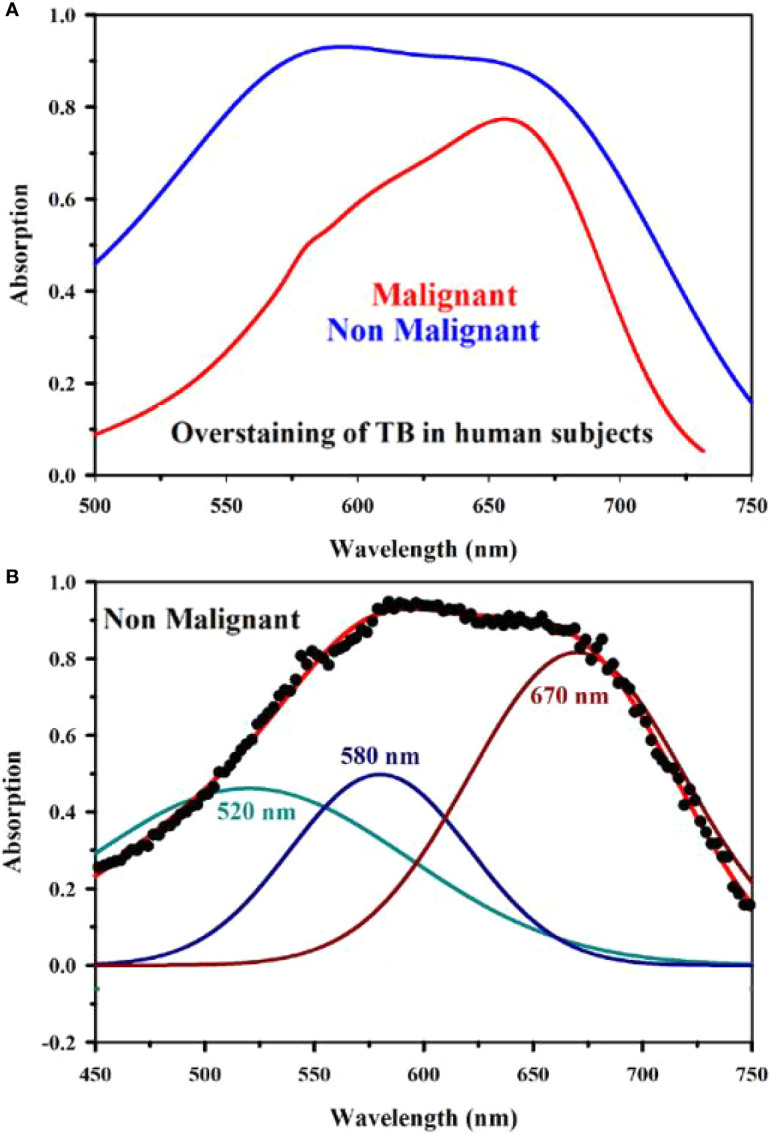
Absorption spectra obtained through “Oral-O-Scope” during a clinical trial on malignant and over-stained non-malignant human oral lesion are shown in **(A)**. The deconvoluted spectra obtained from the fitted curve of over-stained non-malignant lesion are shown in **(B)**. The remarkable absence of monomeric form of TB evident by 610 nm in the nonmalignant overstained lesion is clearly visible.

**Figure 10:**
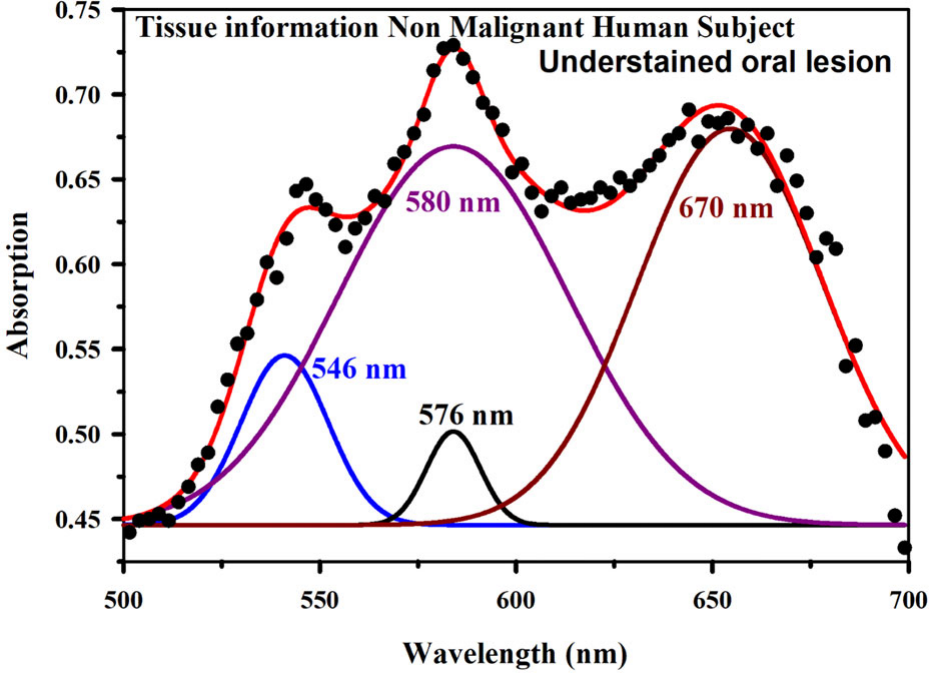
Absorption spectra obtained through “Oral-O-Scope” during a clinical trial on an under-stained non-malignant human oral lesion. The absence of monomeric TB form (610 nm) and presence of tissue information indicated by 546 and 576 nm which are the spectroscopic signature of oxygenated hemoglobin (see text) is clearly visible.

**Figure 8A** shows absorption spectra acquired through our developed “Oral-O-Scope” in a clinical trial on malignant and non-malignant human subjects. It was found that the instrument was able to produce repeatable data under the clinical setting. The spectral deconvolution as shown in **Figures 8B, C** clearly reveals absence of monomeric form of TB (610 nm) in non-malignant lesion (**Figure 8B**) compared to that in malignant lesion **Figure 8C**. The presence of monomeric TB associated with tetrameric forms (520, 580 nm and 670) is distinctly evident in the malignant tissue. Corresponding confirmatory biopsy (histopathological slide) image of the TB stained tissue from the malignant subject is shown in **Figure 8D**. The provisional diagnosis suggested solitary bit of soft tissue from the left buccal mucosa. Histopathological evaluation revealed hyperplastic squamous epithelium with intact basement membrane. However, at one place an invasion of islands of dysplastic epithelium with cellular changes and nuclear hyper-chromatism were observed along with few keratin pearl formations and 1 or 2 abnormal mitoses. High power field of microscope shows highly cellular and vascular tissue stroma as shown in **Figure 8D**. This is suggestive of ‘well-differentiated’ squamous cell carcinoma. We have also explored the possibility of detection of overstaining of oral lesion, which often led “false-positive” in the visual interpretation of TB-staining (32, 35, 36). **Figure 9A** shows absorption spectra acquired through “Oral-O-Scope” in a clinical trial on malignant and over-stained non-malignant human oral lesion. The corresponding deconvoluted spectra are shown in the **Figure 9B**. Note the remarkable absence of monomeric form of TB in the nonmalignant overstained lesion. The understating of oral tissue is found to be detected by our developed “Oral-O-Scope”. **Figure 10** shows absorption spectra acquired through “Oral-O-Scope” in a clinical trial on an under-stained non-malignant human oral lesion. Note the absence of monomeric TB form (610 nm) and presence of tissue information represented by 546 and 576 nm which are the signature of oxygenated hemoglobin (37). **Table 1** summarizes the individual contributions of all the deconvoluted peaks in percentage for all the acquired data in this work. We have tested our device with various forms of inflammations and traumatic ulcers only to find no presence of monomeric form of TB (610 nm) indicating non malignancy in the tissue. The proposed device showed promising potentiality to determine the molecular existence of various forms of TB interactions with DNA. The competitive market analysis and scientific literature showed the existence of few light-based devices which rely primarily on imaging and produce qualitative analysis and hence are only useful as a screening tool. Oral-O-Scope on the other hand uses absorption spectroscopy and subsequent deconvolution of obtained peaks to ascertain the presence of TB in various forms. **Table 2** represents a comparative representation of the commercially available devices vis-à-vis Oral-O-Scope in terms of various measurement parameters. The commercially available instruments available in open market are dependent largely on imaging (Chemiluminescence, Autoﬂuorescence, and Fluorescence) of tissues which provide no insight on the variety of forms of the presence of TB in tissues. Oral-O-Scope, on the contrary measures data and analyses the presence of TB in various forms confirmed by their spectroscopic signatures using reflectance spectroscopy.

**Table 1:** Weightage of the deconvoluted spectra of TB sample in various conditions.

Sample (TB in)	Spectral Contributions (%)								Forms of TB
	520 nm	546 nm	550 nm	576 nm	580 nm	610 nm	640 nm	670 nm	
Water	-	-	-		32.64	33.05	34.3	-	Monomer+ Dimer
Protein (HSA), pH=7	-	-	-		15.3	49.67	35.01	-	Monomer+ Dimer
Protein (HSA), pH=2	-	-	-		33.6	39.4	26.96	-	Monomer+ Dimer
Protein (HSA), pH=11	-		66.79		-	33.2	-	-	Monomer+ Trimer
TB-DNA pH 7.4	-	-	-		26.54	43.51	30.13	-	Monomer+ Dimer
Normal cell (HEK)	-	-	-		34.38	0	65.61	-	Dimer
Lung cancer cell (A549)	-	-	-		66.33	13.15	20.50	-	Monomer+ Dimer
Non-malignant oral lesion	36.16	-	-		21.91	0	-	42.95	Tetramer only
Malignant oral lesion	19.39	-	-		0.78	49.9	-	29.87	Tetramer+ Monomer
Overstained malignant oral lesion	7.06	-	-		00.24	58.54	-	34.14	Tetramer+ Monomer
Under-stained non-malignant oral lesion	-	8.18	-	2.79	48.17	-	-	40.84	Oxygenated Hemoglobin+Tetramer

Note that the individual contribution of monomeric form (610 nm) of TB is substantially high in cells with malignancy and hence can be used as a suitable marker for oral cancer detection.

**Table 2:** Comparison of commercially available devices with Oral-O-Scope.

Device	Vizilite & Vizilite Plus	Velscope	Identaﬁ	Microlux/DLTM	Oral-O-Scope
Measurement Principle	Chemiluminescence Imaging	Autoﬂuorescence Imaging	Fluorescence Imaging	Chemiluminescence Imaging	Absorption Spectroscopy
Type of Lesion	PMD, OSCC, OL	PMD, OSCC	OSCC	OSCC	PMD, OSCC, OL
Analysis Type	Qualitative	Qualitative	Qualitative	Qualitative	Quantitative
Screening/ Diagnostic	Screening	Screening	Screening	Screening	Diagnostic
Sensitivity	Low	High	Low	High	High
Specificity	Low	High	High	Low	High
Molecular Identification	No	No	No	No	Yes

It is evident that Quantitative representation and quick determination of Lesion type gives Oral-O-Scope distinct advantage over the others.

## Conclusion

The present work is to study the molecular interactions of a cationic dye TB with protein and DNA to understand the efficacy of the dye for the diagnosis of oral cancer in human subjects. While protein (HSA) is found to be almost noninteracting with the dye TB in physiological condition, genomic DNA shows affinity toward the dye in the similar experimental condition, which could be the key for labeling malignant cell containing more nucleic material in the cytosol. We have shown the existence of monomeric form of TB dye (absorption peak at 610 nm) in DNA associated with some dimeric form of the dye. The FRET studies also confirmed strong interaction of TB with genomic DNA at the molecular level. In *ex-in vivo* studies on normal (HEK) and cancer (A549) cell lines reveal consistent observation in the line of the *in vitro* studies on TB with genomic DNA. While cancer cells can retain TB in monomeric form (610 nm) along with some percentages of dimer, normal can *only* hold aggregated form of the dye. Our further studies on the clinical trial also confirm that existence of monomeric TB in the cancer lesion with some aggregated form can be used to identify malignancy in the oral lesion. This contrasts with visual or colorimetric method of evaluation where the difference of inflammation induced atypia with tobacco-induced malignancy is not clear. The molecular level diagnosis by Oral-O-Scope enables us to understand the interaction of TB with proliferated DNA which is absent in in any inflammation induced atypia. We have also identified over staining leading to conventional false positive cases in the visual examination based on dye aggregation behavior in the oral tissue. We are confident that the device and the methodology (spectral deconvolution) used in the report can also be use useful for the class and degree of the malignancy of the oral lesion through large scale clinical trial, which is under way in our group.

As a pilot study, the sample size is appropriate for ascertaining the functioning of the instrument and the probe molecule. A larger clinical validation with a higher number of patients will be performed in future. The degree of malignancy is a promising possibility through Oral-O-Scope by application of various statistical models over the collected data in significant numbers as the initial data proves the efficacy of the device in converting malignancy cells in numerical numbers.

## Data Availability Statement

The datasets generated for this study are available on request to the corresponding author.

## Ethics Statement

The studies involving human participants were reviewed and approved by The Ethical Committee, Awadh Dental College & Hospital, Jamshedpur, India. The patients/participants provided their written informed consent to participate in this study. Written informed consent was obtained from the individual(s) for the publication of any potentially identifiable images or data included in this article.

## Author Contributions

SS was involved in planning of the study, development of software, and overall coordination, data collection, checked the final data and reviewed the manuscript. AH was involved in design and development of instrument and preparation of initial draft. OS was involved in data collection both in laboratory and hospital-based field trial. NC contributed by doing bio-chemical experimentation. TC contributed by doing microscopy based and cell culture experimentation. AA was involved in analysis of the data and revised the manuscript. PS contributed by doing all FRET based experimentation and related data analysis. RG performed spectroscopic investigations during the revision process. DS and AB was involved in all fiber optics-based development necessary for the experimentation. PM and AM revised the manuscript. MB planned the cell culture experiments and revised the manuscript. SB supervised and planned the clinical trial activities. SA, AAl, and AH reviewed the study planning, checked the final data, and revised the manuscript. SKP conceptualized and planned the study, interpreted the data, and revised the manuscript. All authors contributed to the article and approved the submitted version.

## Conflict of Interest

Authors PM and AM were employed by the company Ezerx Health Pvt. Ltd.

The remaining authors declare that the research was conducted in the absence of any commercial or financial relationships that could be construed as a potential conflict of interest.

The handling editor declared a past co-authorship with the authors.
